# Supplementary material for: Analyses of the Genetic Diversity and Population Structures of Sporothrix spp. Clinical Isolates from Paraíba, Brazil
Source: J Fungi (Basel). 2024 Dec 9;10(12):848. doi: 10.3390/jof10120848 (PMC11678700; doi:10.3390/jof10120848)
Supplement: Supplementary file 1 [file jof-10-00848-s001.zip › jof-3265006-supplementary.pdf]

Table S1. Polymorphic statistics calculated for the combinations #3EcoRI-GA/MseITT and #5 EcoRI-GA/MseIAG for *Sporothrix* spp.

#3 EcoRI-GA/MseI-TT

**Species**	**Isolates (n)**	**Fragments**	**H**	**PIC**	**E**	**Havp**	**MI**	**D**	**Rp**
S. brasiliensis	46	43	0,4736	0,3614	16,5652	0,0002	0,0039	0,8517	7,3913
***S. brasiliensis* PB**	38	22	0,4294	0,3372	15,1315	0,0005	0,0077	0,5271	2,6842
S. schenckii	6	52	0,4838	0,3668	21,3333	0,0015	0,0330	0,8324	27,3333
S. globosa	6	45	0,4999	0,3749	22,6666	0,0018	0,0419	0,7472	22,6666
**Overall**	58	92	0,3108	0,2625	17,7069	0,000005	0,0010	0,9629	17,8275

#5 EcoRI-GA/MseI-AG

**Species**	**Isolates (n)**	**Fragments**	**H**	**PIC**	**E**	**Havp**	**MI**	**D**	**Rp**
S. brasiliensis	46	45	0,4905	0,3702	19,4130	0,0002	0,0046	0,8140	7,1739
***S. brasiliensis* PB**	38	22	0,2788	0,2399	18,3157	0,0003	0,0061	0,3070	4,0000
S. schenckii	6	51	0,4992	0,3746	24,5000	0,0016	0,0399	0,7700	25,0000
S. globosa	6	52	0,4959	0,3729	23,6666	0,0015	0,0376	0,7936	27,3333
**Overall**	58	86	0,3614	0,2961	20,3620	0,000007	0,0014	0,9439	18,9310

The marker characteristics for the primer combinations used in the AFLP reaction are provided in Table S1. The PIC (polymorphic information content) value for the *Sporothrix brasiliensis* PB isolates ranged from 0.2399 to 0.3372, demonstrating equivalent levels of polymorphic information content, which revealed the excellent ability of each primer combination to detect intra- and interspecific polymorphisms.

Discriminatory power (D) is considered the probability that two random individuals will present a different band pattern. All markers demonstrated high overall discriminatory power (D: 0.9439-0.9629). In contrast, analyzing the values in the *S. brasiliensis* PB isolates, lower discriminatory power values (0.3070-0.5271) were observed compared to the general *Sporothrix brasiliensis* group, indicating lower diversity among the isolates from Paraíba.

The Marker Index (MI) was calculated as the product of the effective multiplex ratio (E) and the average expected heterozygosity (Havp) for polymorphic markers. The result was used to estimate the usefulness of each marker system. Generally, MI values ranged from 0.0010-0.0014 for both combinations. The resolution power (Rp), which is the ability of each primer combination to detect the level of variation among individuals, was highest in the primer combination #5 (Rp = 18.9310).

The expected heterozygosity (H), described as the probability that an isolate will be heterozygous for the locus in the population, was also calculated. The average heterozygosity for *Sporothrix brasiliensis* species ranged between 0.4736-0.4905 (Table S1). When comparing the results for *Sporothrix brasiliensis* Pb isolates, high heterozygosity was identified with the marker #3 EcoRI-GA/MseI-TT 0.4294, confirming that the marker revealed cryptic diversity.
